# Persuasion ability in children from 6 to 12 years old: Relations to cognitive and affective theory of mind

**DOI:** 10.3389/fpsyg.2022.966102

**Published:** 2022-09-15

**Authors:** Carmen Barajas, María-José Linero, Rafael Alarcón

**Affiliations:** ^1^Department of Developmental and Educational Psychology, University of Málaga, Málaga, Spain; ^2^Department of Psychobiology and Methodology of Behavioral Science, University of Málaga, Málaga, Spain

**Keywords:** persuasion, cognitive theory of mind, affective theory of mind, school age, normotypical development

## Abstract

This study analyzes the relation between cognitive and affective components of theory of mind (ToM) in school-aged children and persuasion abilities. One-hundred forty-three normotypical school children aged 6 to 12 were administered cognitive and affective ToM tasks and one persuasion production task. A set of regression models showed that only the affective ToM component can predict both the persuasion total scores and all its indicators' scores. Children with a greater ability to attribute emotional mental states do not only produce a wider variety of persuasive arguments but also arguments focused on the persuadee and those with mental-related content. Both Hidden Emotion and Belief-Emotion (negative) tasks have been predictive of persuasion total scores. This study provides data on specific contribution of cognitive ToM and affective ToM on indicators of variety and quality of persuasive arguments independently.

## Introduction

### Persuasion ability and ToM

This article studies the persuasion ability in children aged from 6 to 12 and its relation to theory of mind (ToM), considering the cognitive, and the affective ToM separately.

Persuasion consists of the pragmatic use of language, oral or written, to change other people's minds; it is an expression with the aim of making others think, wish, feel or say something that makes them perform or inhibit a given action (To et al., 2016). Lonigro et al. (2018) evinced that persuasive essays are mainly based on syntactic (subordinate clauses) as well as semantic (mental state terms) specific language resources. In fact, the ability to persuade requires an understanding of both how mental states influence behavior and the way those mental states can be influenced and altered through language. Therefore, the ability to persuade requires not only language competences but also ToM abilities that allow considering the persuadee's mental states (epistemic and/or emotional).

The relation between persuasion ability and ToM abilities is so close that the indicators of persuasion ability are often measures of the ability to consider mental states, since the efficiency of persuasion lies in applying mind understanding to persuasive purposes. Thus, persuasion ability in children has been primarily analyzed on the basis of two variables: variety and quality of the arguments produced, both indicators for the ability to place oneself in the persuadee's mental perspective.

Regarding the variety of produced arguments, various studies have linked ToM abilities to the number of arguments that the participants produce in a persuasion task. The study conducted by Slaughter et al. (2013) involved children aged from 3 to 8 in an interactive task in which they were expected to persuade a hand puppet (peer) to eat some broccoli or brush their teeth. Up to three times, the puppet held a belief that justified their refusal to do what was being proposed. The results showed that the scores in False Belief tasks are significantly connected to the number of different produced persuasive arguments, which was considered as an indicator of flexibility to generate counters to character's mental perspective.

To et al. 2016 work also provides information about the level of persistence or the number of attempts that children aged from 6 to 12 deploy in a task in which they were allowed to persuade an adult up to five times. The authors point out that persistence deals with understanding the possibility to influence the persuadee's mind, even when it could depend as well on the persuadee's degree of interest in the object. On the other hand, Kołodziejczyk and Bosacki (2016) found that children aged from 5 to 7 produced a higher number of persuasive arguments when they were tasked to persuade an adult (asymmetrical interaction) than when they interacted with a peer (symmetrical interaction).

Other works have assessed the relation between persuasion and ToM on the basis of quality of the arguments produced. In Bartsch and London's study (Bartsch and London, 2000), children aged from 4 to 12 were urged to select the most effective persuasive argument for making a character persuade their mother to buy them a pet or a toy. In a later study (Bartsch et al., 2007), children aged from 3 to 7 were narrated a story in which the main character refused to do something and held a belief that justified their refusal. Results from both studies revealed a relation between the performance in ToM tasks and the ability to select the argument that supported the persuadee's opinion. On their side, Slaughter et al. (2013) considered as a genuine persuasive argument every verbal resource other than simply ordering or begging the character to do the action. They identified increasing levels of quality in the arguments, ranging from mere non-elaborated orders or expressions of politeness to more elaborated arguments intended to change the persuadee's initial mental state, such as offering an incentive, modeling, or encouraging to cognitively rethink the situation.

Given the evidence that the key to persuasion lies in considering the mental perspective of the person to be persuaded (for both the persuadee's benefit-selfish persuasion-and the persuader's benefit-prosocial persuasion-), some studies have drawn an explicit distinction between the arguments addressed to the persuader (self-oriented) or to the person to be persuaded (hetero-oriented) within their category systems for persuasive arguments. Children who produce self-oriented arguments only consider their own point of view. Therefore, they fail to address the reasons that the persuadee opposes, whereas children who use hetero-oriented arguments try to take into account others' points of view as well (Sato and Wakebe, 2012; Kołodziejczyk and Bosacki, 2015, 2016).

In this line of research, To et al. (2016) involved children aged from 6 to 12 in two role-play tasks in which they were urged to persuade their mother to buy them a cat or to convince the experimenter to allow them to play with his/her iPhone. The authors proposed a code system for persuasive arguments that consists of four levels of quality based on their consideration of the persuader's or the persuadee's needs and interests. At the lowest level, they placed those responses that fail to imply an intention of persuading or the ones that probably end up in refusal. At the highest level, they ranked expressions aimed at the persuadee's interests or benefits. The results demonstrated that the children with the highest scores in ToM tasks produced higher-level persuasive strategies.

Based on this same distinction, Perucchini et al. (2003) found that the use of hetero-oriented strategies increases with age, and that it is connected to the understanding of first-order false belief, contrary to self-oriented strategies. In a more recent study, using a selfish persuasion task with children aged from 8 to 11, Lonigro et al. (2017) observed that scores in cognitive ToM were positively associated with hetero-oriented strategies and negatively with self-oriented strategies, regardless the age.

Alongside with this distinction between self-oriented and hetero-oriented arguments, it might be relevant to differentiate whether the arguments are focused on material or mental aspects. When the arguments the children provide themselves or those that they are given to pick from are analyzed in detail, it is possible to confirm that some are focused on material or physical aspects (“I'll give you some candies if you let me play with it”), whereas others are focused on mental aspects (“Do not worry about your iPod, I'll be very careful”). Although no study makes a distinction between these two arguments scoring them separately, it is logical to hypothesize that a higher ToM ability can result in a higher attention or consideration to more mentalistic than material aspects, contributing to produce arguments with the purpose of changing the persuadee's mental states.

In short, although the majority of studies on the relationship between ToM and children's persuasion abilities have considered the indicators of variety and quality of the persuasive arguments, not many of them have simultaneously included both measures. Moreover, regarding the quality of the arguments, the most frequent criterion has been whether the arguments were focused on the persuadee or the persuader, but no studies considering whether the arguments were focused on material or mental aspects have been found. The aim of this study is to assess persuasion ability, considering both variety and quality of persuasive arguments, according to the self-oriented vs. hetero-oriented and material vs. mental criteria.

### Cognitive and affective ToM

All the mentioned studies find a relation between ToM and persuasion measures, both prosocial and selfish. The ToM measures many of them use are primarily based on False Belief or intentionality understanding tasks (Bartsch and London, 2000; Bartsch et al., 2007; Kołodziejczyk and Bosacki, 2015, 2016; To et al., 2016). Some studies that introduce other ToM tasks have also found a relation between ToM total scores and the persuasion ability, such as Slaughter et al. (2013), that introduces a Belief-Emotion task; Peterson et al. (2018), that include Hidden Emotion and Sarcasm; or Lonigro et al. (2017), that uses some of the Strange Stories by Happé (1994).

Notwithstanding, it is inferred from the category systems for produced persuasive arguments and results that effective persuasion requires attributing not only epistemic but also emotional mental states. For instance, Bartsch’s studies (Bartsch and London, 2000; Bartsch et al., 2007, 2010, 2011) show how competent persuaders can use complex reasoning about mental states when building a persuasive argument. Children were able to reason about links between desire and belief (“I'll say that I want to eat broccoli so they think that it's delicious”) or between belief and emotion (“I'll say to them that I'll have to throw it away if they do not eat it, and that's a pity, when there are so many children that have no food”). Similarly, To et al. (2016) grant higher scores to persuasive arguments that consider the persuadee's emotional needs or worries (“I'll handle it carefully”). On the other hand, Kołodziejczyk and Bosacki (2016) and Lonigro et al. (2017) distinguish within the pesuadee-oriented arguments (hetero-oriented) a subcategory for emotion-targeted arguments, i.e., introducing changes to the persuadee's emotional state, such as guilt or pity. In this sense, it is appropriate to consider a difference between the ability to attribute epistemic mental states (cognitive ToM) and emotional mental states (affective ToM).

A distinction between cognitive ToM and affective ToM has been drawn on the basis of cognitive neuroscience studies (Sebastian et al., 2012; Dvash and Shamay-Tsoory, 2014), that have recognized different developmental patterns throughout childhood for each one. The development of advanced affective ToM abilities is later than the cognitive component development. Consequently, the ability to attribute second-order false beliefs is acquired by the age of 6–7 (or even earlier if, as Lombardi et al., 2018 suggest, it is considered that some answers to the questions of the task are correct at an inferential level, which fail to meet the researcher's expectation, however), whereas the ability to attribute a second-order emotional-belief (e. g. “she thinks he feels”) or to attribute emotional states related to states of knowledge (e. g. “she'll be happy because she will think that there are candies inside the box, she does not know there are medicines”) is acquired later. By using specific affective ToM measures, Slaughter et al. (2013) found that the scores in the Belief-Emotion task are related to the variety of prosocial persuasion arguments in children aged from 3 to 8. Peterson et al. (2018) found as well that the scores in the Hidden Emotion task are also related to the variety of prosocial persuasion arguments in children aged from 3 to 11, and that the scores in the Hidden Emotion and Sarcasm tasks are also related to the variety of persuasion arguments in children aged from 5 to 12. Nonetheless, in this last case it is a very challenging selfish persuasion task since the persuader's goal is to obtain a personal benefit at the persuadee's expense.

Given this evidence on the relation between emotions understanding and the ability to persuade, it seems appropriate to study in depth the role that affective ToM may play in persuasion ability; it is possible that the cognitive and affective components of ToM impact differently on persuasion ability.

In short, the studies on the relation between ToM and children's persuasion abilities have used different ToM measures, mainly False Belief and intentionality understanding tasks (cognitive ToM), albeit some have included emotions attribution measures (affective ToM). Nevertheless, it has not been found any record of studies that analyze the specific contribution of cognitive ToM and affective ToM independently through an overall measure for each of the two ToM components. This study aims to analyze the specific contribution of cognitive ToM and affective ToM to children's persuasion ability, as well as the specific contribution to the variety and to the quality of persuasive arguments.

The main aim of this study is to analyze the relation between ToM abilities and persuasion ability of children aged from 6 to 12. The specific aims are: (1) to analyze the specific contribution of cognitive ToM and affective ToM to the ability to produce persuasive arguments; and (2) to analyze the contribution of ToM abilities to two different persuasion indicators: the variety of produced arguments and their quality, according to whether the arguments are self-oriented or hetero-oriented, and focused on material or mental aspects.

## Materials and methods

### Participants

The study was carried out with a sample of 143 primary school students (76 boys and 67 girls) aged 6–12 years old (*M* = 9.34, *SD* = 1.73). The participants were distributed in six class groups that belonged to three public schools from the same city district. All children were administered the Peabody Picture Vocabulary Test-III (Dunn et al., 2006), so only children with normotypical development were part of the sample. Table 1 shows the mean (*M*), standard deviation (*SD*) of the age variable and the frequency distribution of the sex variable in each of the courses.

**Table 1:** Mean (*M*) and standard deviation (*SD*) descriptive of the age variable and frequency distribution of the gender variable by grade.

Grade	Age		Gender	
	*M*	*SD*	Boy	Girl
First	6.80	0.32	12	13
Second	7.87	0.27	10	10
Third	8.87	0.32	11	13
Fourth	9.78	0.31	13	13
Fifth	10.73	0.42	14	8
Sixth	11.75	1.73	16	10

### Measures

A persuasion task and a set of ToM tasks were administered.

#### Persuasion task

A persuasion production task was designed drawing on the persuasion understanding task from the “Strange Stories” battery (Happé, 1994). Happé's task consists of a story in which a boy lies to his school canteen's cook by telling him that he would not have any dinner at home that night, so he asks the cook to double his portion of sausages, which is his favorite food. The participant is asked whether what the character says is true or not, as well as to explain why he says that; therefore, the task measures the ability of the participants to attribute the character a motivation of igniting a sense of pity in the cook by creating a false belief idea to achieve a personal benefit.

The task that has been designed consists of a brief story in which the participant is the main character and is assigned a situation in which they must produce persuasive arguments that relate to a given motivation, which is also assigned to him/her as follows: “You are going to your friend's birthday celebration, and you realize that they brought your favorite cake for this party. Everyone is to receive one slice only. When your friend's mother finishes cutting the cake, you see that there is a slice left. You have already eaten one. What would you say to your friend's mother to convince her to give it to you?” Once the participant had provided their first answer, the experimenter, role-playing a mother, asked them for a new argument, and this was repeated up to three times, following Slaughter et al. (2013) and Peterson et al. (2018) procedure. This task assesses the ability to develop persuasive arguments aimed at achieving one's own benefit.

*Coding and scoring.* Two indicators of persuasion ability have been considered: the variety and the quality of the arguments.

*Variety*. 1 point is awarded for each different persuasive argument (0 to 3 points).

*Quality*. Each of the three arguments provided by each participant is classified according to the following coding system, meaning that the total quality score for a persuasive argument ranges from 0 to 4 points.

*Expressions that are not persuasive*: 0 points (“I would not tell her anything,” “I do not care if she does not give it to me”).

*Simple requests or expressions of politeness*: 0.5 points (“give it to me,” “please, can you give it to me?”).

*Persuasive arguments*: Any expressions targeted to the persuadee that include a reason to convince them. Each argument is rated considering two quality indicators simultaneously: self-oriented vs. hetero-oriented and material vs. mental focused.

Self-oriented vs. hetero-oriented. 1 point: an argument focused on a personal need or desire (“it is the cake that I like the most,” “I am very hungry,” “my mother is not going to serve dinner for me”); 2 points: an argument that focuses on the individual being persuaded or on a third party (“because I am your son's best friend,” “if no one is going to eat it, you will have to throw it away,” “you have made the richest cake I have ever tasted”).

Material vs. mental. 1 point: an argument that focuses on material gain (“my mother is not going to serve dinner for me,” “if no one is planning to eat it, you will have to throw it away”); 2 points: an argument focusing on a consequence or mental benefit (“it is the cake that I like the most,” “you are an excellent cook”).

To calculate persuasion ability scores, it has been used for each participant the statement with the highest score in terms of quality.

*Total persuasion ability score*. Sum of the variety score and the total quality score (0 to 7 points).

#### ToM tasks

A cognitive ToM score and an affective ToM score were obtained. Cognitive ToM score was obtained from the False Belief about Location of Second-Order task (Núñez, 1993) and cognitive questions of Faux Pas tasks (Baron-Cohen et al., 1999; Zalla et al., 2009). Affective ToM score was obtained from the Belief-Emotion (negative valence) task (Harris et al., 1989), and Hidden Emotion task (Harris et al., 1986).

*Second-Order False Belief about Location* (Núñez, 1993). It assesses the ability to attribute someone a false belief about a third person's knowledge of an object's location. Sally puts her marble in her basket and leaves the room; meanwhile, Anna moves Sally's marble into her box without realizing that Sally is watching. The participant is asked where Anna thinks that Sally would look for her marble, as well as to justify their response.

*Coding and scoring*. 0 points: no second-order reasoning (“in the box, because her marble is there”); 1 point: second-order reasoning by implicitly referring to the two mental states (“in the basket, because she has not seen that Sally was watching”); 2 points: an explicit reference to one of the two mental states (“in the basket, because Anna does not know that Sally was watching”); 3 points: an explicit reference to the two mental states (“in the basket, because Anna thinks that Sally does not know what has happened”).

*Faux Pas* (Baron-Cohen et al., 1999; Zalla et al., 2009). It tests the ability to understand that a character who does not know certain information can say something that unintentionally produces a negative emotion in another person. Two Faux Pas stories were administered. In each task, the following questions have been posed: (1) the *detection* question. “In the story did anyone say something they should not have said or something awkward?”; (2) the *person identification* question*: “*Who said something they should not have said or something awkward?”; (3) the *content* question: “What did they say that they should not have said or what was it awkward?”; (4) the *explanation* question: “Why should not they have said it or why was it awkward?”; (5) the *false belief* question [This question was different for each story]: *“*Did they know/remember that…?”

In this study the answers to Explanation Question and False Belief Question have been taken into account jointly to check whether the child understands that the faux pas was a consequence of the speaker's false belief rather than being an action with malicious intent.

*Coding and scoring*. 0 points: no attribution of a false belief to the speaker (“because you must not speak ill of someone”); 1 point: implicit attribution of ignorance to the speaker that makes the blunder by referring to the effect produced in the person that receives the harm (“because the girl that is in the toilet would feel bad”); 2 points: allusion to the perspective of the person that makes the blunder (“because they do not know if the girl is in the toilet and can listen to what they are saying”).

*Negative Belief-Emotion* (Harris et al., 1989). It assesses the ability to attribute to another person a negative emotion caused by a wrong belief. The participant is shown a box of medicines that actually contains sweets, and is asked how the character will feel when he/she receives the box without opening it, and why.

*Coding and scoring*. 0 points: no attribution of a negative emotion (“he/she will be happy, because he/she loves sweets”); 1 point: attribution a negative emotion by referring to the supposed content (“sad, because no one likes medicines”); 2 points: an explicit reasoning about the mental state (“disillusioned, because he/she does not know that actually there are sweets inside”); 3 points: an explicit mentalistic reasoning (“sad, because he/she will believe there are medicines”).

*Hidden Emotion* (Harris et al., 1986). This task assesses the skill to understand that a person can feel one emotion but feign another. The participant is told the story about a character that feels sad after being mocked by their classmates, and who tries to hide his feelings so they stop mocking him. The student is asked how the character tries to look (choosing a neutral, a sad, or a happy face), and why.

*Coding and scoring*. 0 points: choosing the sad face; 1 point: choosing the neutral or happy face and alluding to the behavioral consequence the character tries to avoid (“so that they do not call him baby”); 2 points: an implicit reference to hide the emotion (“so that they do not see he is sad”); 3 points: providing an explicit mentalistic justification (“so that they do not know he is sad”).

Cognitive ToM score ranges from 0 to 7 points. Affective ToM score ranges from 0 to 6 points. And it was also calculated a total ToM score ranging from 0 to 13 points.

### Procedure

The present study was carried out in three public schools. After contacting the schools, explaining the objective of the research and obtaining families' consent to undertake the study, the classes were organized and the tests were administered, in two 30-min sessions. The first session consisted of administering the ToM tasks while the second session was dedicated to persuasion tasks.

### Data analysis

A preliminary descriptive analysis has been carried out with all the variables measured in this study. Next, a linear correlation analysis calculating Pearson coefficient was performed between the variables total score in persuasion, total score in ToM and age. Based on the results, a multiple linear regression analysis has been carried out for the prediction of the persuasion total score (response variable), the cognitive ToM and affective ToM dimensions (predictor variables), and the age variable as a covariate. Subsequently, four multiple linear regression analysis have been carried out being the variety score, quality of the arguments total score, self-oriented vs. hetero-oriented arguments score and material vs. mental arguments score response variables, and the cognitive ToM and affective ToM dimensions independent variables, the covariate being the age variable. Finally, a multiple linear regression analysis has been carried out for the prediction of the persuasion total score (response variable), being affective ToM tasks predictor variables, and the age variable as a covariate.

## Results

### Preliminary descriptive analysis

Table 2 shows the results obtained in the descriptive analysis of the persuasion variable (persuasion total score, variety score, quality of the arguments total score, self-oriented vs. hetero-oriented score and material vs. mental arguments score) and ToM variable (ToM total score, cognitive ToM total score, affective ToM total score, cognitive ToM tasks, and affective ToM tasks). The skewness and kurtosis values of all variables are within the range ± 1.96, meaning that the variables follow a normal distribution.

**Table 2:** Mean (*M*), standard deviation (*SD*), observed range (*R*), skewness (*g*1) and kurtosis (*g*2) of persuasion and ToM variables.

			*M*	*SD*	*R*	*g*1	*g*2
Persuasion	Variety		1.27	0.73	0–3	0.29	−0.02
	Quality	Self-oriented vs. hetero-oriented	0.96	0.79	0–2	0.29	−1.46
		Material vs. mental	0.87	0.73	0–2	0.48	−1.10
	Total quality of the arguments		1.86	1.44	0.5–4	0.45	−1.52
	Total persuasion		3.16	1.66	0.5–7	0.40	−0.83
ToM	Cognitive ToM tasks	Second-order false belief	1.44	0.86	0–3	−0.26	−0.70
		Faux Pas: cognitive ToM questions	0.84	0.60	0–2	0.07	−0.32
	Total cognitive ToM		2.29	1.1	0–5	−0.23	−0.56
	Affective ToM tasks	Belief-Emotion (negative)	1.16	0.73	0–3	1.56	2.35
		Hidden Emotion	1.76	1.06	0–3	−0.03	−1.41
	Total affective ToM		2.92	1.38	0–6	0.26	−0.43
	Total ToM		5.19	1.96	1–10	0.01	−0.38

### Relationships between age, ToM total score, cognitive ToM total score, affective ToM total score, and persuasion total score

The results showed that the relation between age and ToM total score is positive and statistically significant [*r_xy_* = 0.208, *p* < 0.05], and also that there is relation between ToM total score and persuasion total score [*r_xy_* = 0.256, *p* < 0.005], that is, an increase in age is related to an increase in the total score in ToM; and an increase in the total score in in ToM is related to an increase in the total score in persuasion. The correlations of age and total score in persuasion was not significant in Table 3.

**Table 3:** Linear correlations between age, ToM total score, cognitive ToM total score, affective ToM total score, and persuasion total score.

	Age	Persuasion total score	ToM total score	Cognitive ToM total score
Persuasion total score	0.05			
ToM total score	0.22^**^	0.27^**^		
Cognitive ToM total score	0.05	0.08	0.73^**^	
Affective ToM total score	0.27^**^	0.32^**^	0.84^**^	0.24^**^

** *p <* 0.01.

As for the specific correlations of each of the ToM components with age and persuasion, the results showed that affective ToM evinces a positive and statistically significant correlation with age [*r_xy_* = 0.27, *p* < 0.01] as well as with persuasion [*r_xy_* = 0.32, *p* < 0.01]. However, the correlations of cognitive ToM total score and both age and persuasion were not significant.

### Multiple linear regression analysis with persuasion total score (response variable), cognitive ToM and affective ToM dimensions (independent variables), the covariate being the age variable

The results showed that the affective ToM is the only significant predictor of the total score in persuasion [*F* (3, 139) = 5.16; *MSE* = 67.59; *p* < 0.005]. Of the variables in the equation, only the affective ToM proved to be statistically significative; it can be indicated that for each increase in one point in the affective ToM variable there is an increase of 0.881 points in the persuasion variable. The results of the fitted model are shown in Table 4.

**Table 4:** Results of the multiple linear regression of the total score in persuasion (response variable) and the cognitive ToM and affective ToM dimensions (independent variables), the covariate being the age variable.

	*β*	*t*	*p*
Intercept	4.06	2.32	0.022
Cognitive ToM	0.03	0.10	0.918
Affective ToM	0.88	3.76	0.000
Age	−0.09	−0.48	0.634

As only the affective ToM has proved to be a predictor variable of the persuasion total score, a multiple linear regression analysis has been carried out to determine the specific contribution of each of the considered affective ToM tasks, with persuasion total score (response variable), affective ToM tasks (predictor variable), and the age variable as a covariate.

The results showed that the adjusted model includes both Belief-Emotion (negative) and Hidden Emotion tasks [*F* (3, 140) = 5.39; *MSE* = 69.91; *p* = 0.002]. It can be indicated that for each increase in one point in Belief-Emotion (negative) there is an increase of 1.12 points in persuasion total score, and for each increase in one point in Hidden Emotion there is an increase of 0.76 points in persuasion total score. The results of the fitted model are shown in Table 5.

**Table 5:** Results of the multiple linear regression with persuasion total score (response variable), affective ToM tasks (predictor variables), and the age variable as a covariate.

	*β*	*t*	*p*
Intercept	3.97	2.35	0.020
Belief-emotion (negative)	1.12	2.70	0.008
Hidden emotion	0.76	2.53	0.012
Age	−0.08	−0.42	0.678

Based on these results obtained, we will deepen by carrying out multiple linear regression analysis with the indicators of the persuasion variable, variety and quality of the arguments (response variables).

### Multiple linear regression analysis with variety score (response variable), the cognitive ToM and affective ToM dimensions (independent variables), the covariate being the age variable

The results obtained show that the affective ToM variable is the only significant predictor of the variety of persuasive arguments [*F* (3, 139) = 4.99; *MSE* = 2.48; *p* = 0.003]. Of the variables in the equation, only the affective ToM proved to be statistically significative; it can be indicated that for each increase in one point in the affective ToM variable there is an increase of 0.175 points in the variety of the arguments variable. The results of the fitted model are shown in Table 6.

**Table 6:** Results of the multiple linear regression with variety score (response variable) and the cognitive ToM and affective ToM dimensions (independent variables), the covariate being the age variable.

	*β*	*t*	*p*
Intercept	1.07	3.15	0.002
Cognitive ToM	−0.06	−1.15	0.250
Affective ToM	0.18	3.83	0.000
Age	−0.02	−0.49	0.623

### Multiple linear regression analyses with quality of the arguments (response variables), the cognitive ToM and affective ToM dimensions (independent variables), the covariate being the age variable

First, a multiple linear regression analysis was performed with quality of the arguments total score (response variable), the cognitive ToM and affective ToM dimensions (independent variables), the covariate being the age variable. The results showed that for the quality of the arguments total score the model was significant [*F* (3, 139) = 3.79; *MSE* = 7.50; *p* = 0.012]. Of the variables in the equation, only the affective ToM proved to be statistically significative; it can be indicated that for each increase in one point in the affective ToM variable there is an increase of 0.282 points in the quality of the arguments variable. The results of the fitted model are shown in Table 7.

**Table 7:** Results of the multiple linear regression with quality of the arguments total score (response variable) and the cognitive ToM and affective ToM dimensions (independent variables), the covariate being the age variable.

	*β*	*t*	*p*
Intercept	1.05	1.54	0.126
Cognitive ToM	0.04	0.36	0.720
Affective ToM	0.28	3.09	0.002
Age	−0.01	−0.15	0.879

Given this result for the total score of quality of the arguments, a multiple linear regression analysis has been carried out for each of the indicators of quality of the arguments separately.

In regard to the self-oriented vs. hetero-oriented arguments indicator, the results showed that the model was significant [*F* (3, 139) = 6.73; *MSE* = 3.74; *p* = 0.000]. the variables in the equation, only the affective ToM proved to be statistically significative; it can be indicated that for each increase in one point in the affective ToM variable there is an increase of 0.209 points in the self-oriented vs. hetero-oriented arguments indicator score. The results of the fitted model are shown in Table 8.

**Table 8:** Results of the multiple linear regression with self-oriented vs. hetero-oriented arguments indicator (response variable) and the cognitive ToM and affective ToM dimensions (independent variables), the covariate being the age variable.

	*β*	*t*	*p*
Intercept	0.60	1.67	0.098
Cognitive ToM	0.01	0.09	0.923
Affective ToM	0.21	4.32	0.000
Age	−0.03	−0.77	0.446

In regard to the material vs. mental arguments indicators, the results showed that the model was significant [*F* (3, 139) = 4.858; *MSE* = 2.398; *p* = 0.003]. From all the variables in the equation, only the affective ToM proved to be statistically significative; it can be indicated that for each increase in one point in the affective ToM variable there is an increase of 0.166 points in the material vs. mental arguments indicator score. The results of the fitted model are shown in Table 9.

**Table 9:** Results of the multiple linear regression with material vs. mental arguments indicator (response variable) and the cognitive ToM and affective ToM dimensions (independent variables), the covariate being the age variable.

	*β*	*t*	*p*
Intercept	0.61	1.79	0.075
Cognitive ToM	0.01	0.23	0.816
Affective ToM	0.17	3.64	0.000
Age	−0.03	−0.81	0.422

## Discussion

The aim of this study was to explore the relationships between persuasion and ToM abilities (distinguishing between cognitive ToM and affective ToM) in children aged 6 to 12 years. The variety and quality of the arguments were considered as indicators of persuasion ability. Regarding quality it was considered not only if the arguments were self-oriented or hetero-centered, but also if their content was material-related or mental-related. For this purpose, a battery of ToM tasks and a persuasion production task were administered to 143 typically developing children.

To begin analyzing the relationship between persuasion ability and ToM competencies, a Pearson's bivariate correlation analysis was applied considering age, ToM total score and persuasion total score. The results indicate a correlation between age and ToM, and between ToM and persuasion. The relationship between ToM and persuasion constitutes a first global result of this study, which is consonant with those of Bartsch and London (2000), Bartsch et al. (2007), Slaughter et al. (2013), Kołodziejczyk and Bosacki (2015, 2016), and To et al. (2016). All in all, these results reveal that even though age is related to ToM, it is not directly linked to the persuasion ability, so that interindividual differences in persuasion ability appear to be more connected to ToM abilities than with age.

However, these studies consider measures of just cognitive ToM (first and second-order False Belief and intentionality tasks), but fail to acknowledge affective ToM, despite the importance of emotional states attribution in persuasion. We have only found four studies on child persuasion abilities that take measures of both cognitive ToM and affective ToM (Slaughter et al., 2013; those of Lonigro et al., 2017; and study 1 and study 2; Peterson et al., 2018); however, they did not delve into the specific contribution of each ToM component to the persuasion ability, so that, as in our study, they find relationship between the total measure of ToM and persuasion.

To further detail the relationship between affective ToM and persuasion ability, the predictive value of each of the affective ToM tasks on the total persuasion score was investigated through a regression analysis in which both Hidden emotion and Belief-Emotion (negative) tasks were found to be predictors.

Once the relationship between the total measure of ToM and persuasion had been analyzed, we proceeded to segment this relationship into components, both of ToM and persuasion ability. The first step was to investigate which ToM component, cognitive or affective, would have more explanatory weight in the total persuasion score. In a linear regression analysis, affective ToM proved to be the only significant predictor of the total persuasion score.

Once it was verified the relationship between ToM and persuasion and the weight of affective ToM confirmed, the next step was to investigate the impact of ToM on each of the indicators of persuasion: variety and quality of arguments. Regarding variety, in a regression analysis, affective ToM again proved to be the only significant predictor, in this case, of the ability to produce diverse arguments. As for argument quality, in another regression analysis in which the total quality score was included, only affective ToM was significant predictor. Besides, when considering the quality indicators (self vs. hetero-oriented arguments and material vs. mental arguments) separately in two regression analyses, only affective ToM was found to be the only predictor in each of the models, once again.

There are, to our knowledge, only four studies on persuasion and ToM that introduce affective ToM tasks (that of Slaughter et al., 2013; Lonigro et al., 2017 and studies 1 and 2 of Peterson et al., 2018). Slaughter et al. (2013) and study 1 of Peterson et al. (2018) find relationships between persuasion scores (measured as a function of argument variety in a prosocial persuasion task) and affective ToM tasks, specifically Belief-Emotion (positive) and Hidden emotion. That is to say, in prosocial persuasion tasks, the ability to attribute emotional states is related to the ability to produce a diversity of persuasive arguments, namely the ability to consider different mental states in the persuadee. These results are consistent with those of the present study, which also indicate that this relationship (affective ToM-diversity of arguments) can be extended to the case of selfish persuasion.

The latter outcome is in agreement with what Peterson et al. (2018) found in study 2 through a particularly challenging selfish persuasion task (as the aim of persuasion was to obtain a benefit even when it meant detriment to the persuadee). Hence, it seems likely that the attribution of emotional states influences the ability to produce persuasive argument diversity. The results of the present study show that this relationship between affective ToM and argument variety also appears when selfish persuasion is not so demanding, just requiring a benefit for the persuader individual without detriment to the persuadee.

Having said that, it might be asked whether the ability to attribute emotional states has repercussions on the quality of the arguments as well, apart from the variety. Only the study of Lonigro et al. (2017) assesses in a selfish persuasion task the quality of persuasive arguments in relation to both cognitive and affective ToM aspects, through cognitive stories tasks (implying the attribution of cognitive mental states) and emotion and moral stories tasks (implying the attribution of emotional mental states). Nonetheless, unlike the present study, only the cognitive stories (and not the emotion stories and moral stories) measure is finally linked to the quality of persuasive arguments, in particular to the ability to produce hetero-oriented arguments. At this point, it is worth considering the type of affective ToM tasks used in this study and in Lonigro's. Lonigro uses affective ToM tasks through narrations, such as the Strange Stories by Happé (1994), in which the mental states to be attributed have been generated in the characters through language. Meanwhile, in the present study other affective ToM tasks are used; they are tasks in which the mental state to be attributed has been generated in the character through facts or aspects from reality (such as introducing an unexpected object in a container); and this type of tasks shows relation to the persuasion ability in this study.

Therefore, although there seems to be no relation between the understanding of mental states that are generated through language and the ability to persuade (such as in Lonigro et al., 2017), children do seem to use their knowledge of emotional states (assessed through tasks in which emotional states are generated by facts, as in Hidden emotion and Emotion-Belief tasks) in order to produce both prosocial [such as in Slaughter et al., 2013 and Peterson et al.'s study 1 (Peterson et al., 2018)] and selfish [such as in Peterson et al.'s study 2 (Peterson et al., 2018), and this study] persuasive arguments in persuasion situations. Moreover, children seem to use this knowledge of emotional states not only to produce a variety of arguments (such as in Slaughter et al., 2013; Peterson et al., 2018; and this study), but also to elaborate higher-quality arguments as persuadee-oriented arguments, at least upon persuading for selfish purposes (such as in this study). Regarding that relation between affective ToM and the quality of persuasive arguments, this study's data offer an additional contribution: results demonstrate that children use their knowledge of emotional states to produce arguments that are not only aimed at the persuadee (instead of themselves) but also at mental aspects (instead of physical or material aspects).

Perhaps the most divergent result in this study in contrast with previous studies is the relation between the cognitive ToM and the persuasion ability measures. Whereas this relation is found in all the previous studies, in the present study the specific cognitive ToM measure do not relate to the persuasion measures. The age of participants could partially explain these divergences. Slaughter et al. (2013) and Peterson et al.'s study 1 (Peterson et al., 2018) include children aged from 3 and older, and up to 8 and 11, respectively. In these age groups, the performance range in cognitive ToM is higher than in the 6–12 group, which is the case of this study, where there are few participants that obtain low scores in cognitive ToM (considering that second-order cognitive ToM is acquired by the age of 6–7). It may be that, even when cognitive ToM is necessary for persuasion, once cognitive ToM competencies involved in persuasion are achieved the inter-individual differences in the persuasion ability depend on the competency for considering emotional states.

Lonigro et al. (2017) also finds a relation between cognitive ToM and persuasion measures, particularly the ability of children aged from 8 to 11 to produce hetero-oriented arguments in a selfish persuasion task. In the present study (in which a selfish persuasion task and measures of the quality of the arguments depending on whether they are hetero-oriented or self-oriented have been used with children aged from 6 to 12), that relation has not been observed. A possible reason could lie in the fact that the cognitive ToM tests used by Lonigro et al. (2017), that require attributing mental states generated in the characters through language, set a level of demand for which the upper limit is beyond the age of 12 (O'Hare et al., 2009). Meanwhile, in cognitive ToM tests of this kind included in this study (to be exact, the cognitive ToM questions in the Faux pas task) the maximal performance level is reached at an earlier age.

In conclusion, the results of this study are in line with others that establish a relation between ToM and ability to persuade in school aged children; therefore, it provides further information on the ToM components that specifically contribute to the different persuasion ability indicators. Affective ToM seems to be the decisive component in producing both varied and higher-quality arguments: hetero-oriented and mental-focused. These results constitute a contribution to this field of study since, albeit previous studies on ToM and persuasion had used the same mental states attribution tasks, they had not been specifically related to both indicators of variety and quality of the arguments when it comes to persuading for selfish purposes.

Regarding the limitations of this study and future lines of research, it is important to mention that all the data provided by this study are confined to selfish persuasion; the relations observed between ToM components and persuasion measures may be different in the case of prosocial persuasion. Consequently, future studies could look into this comparison. Besides, another variable that could introduce differences in the persuasive arguments produced by children is the symmetry with the interlocutor at whom the persuasion is aimed: an adult or a peer. On the other hand, given the evidence on the relation between emotions attribution and persuasion ability, it would be interesting to assess the emotional valence (positive or negative) of the arguments produced and their relation to affective ToM abilities.

## Data availability statement

The raw data supporting the conclusions of this article will be made available by the authors, without undue reservation.

## Ethics statement

The studies involving human participants were reviewed and approved by Comité Ético de Experimentación de la Universidad de Málaga (Universidad de Málaga). Written informed consent to participate in this study was provided by the participants' legal guardian/next of kin.

## Author contributions

CB and M-JL contributed to conception and design of the study, collected data, organized the database, and wrote the manuscript. RA performed the statistical analysis. All authors contributed to manuscript revision, read, and approved the submitted version.

## Funding

This study was funded by a grant from the University of Málaga (ID B3-2019-02).

## Conflict of interest

The authors declare that the research was conducted in the absence of any commercial or financial relationships that could be construed as a potential conflict of interest.

## Publisher’s note

All claims expressed in this article are solely those of the authors and do not necessarily represent those of their affiliated organizations, or those of the publisher, the editors and the reviewers. Any product that may be evaluated in this article, or claim that may be made by its manufacturer, is not guaranteed or endorsed by the publisher.
